# Supplementary material for: Strengthening regulation for medical products in Tanzania: An assessment of regulatory capacity development, 1978–2020
Source: PLOS Glob Public Health. 2024 Oct 16;4(10):e0003241. doi: 10.1371/journal.pgph.0003241 (PMC11482716; doi:10.1371/journal.pgph.0003241)
Supplement: Attachment — Submitted filename: Strengthening regulation Tanzania Feb2024.docx [file pgph.0003241.s001.docx]

PLOS Global Public Health

Strengthening Regulation for Medical Products in Tanzania: An assessment of Regulatory Capacity Development, 1978–2020

Thank you for the opportunity to comment on this interesting paper. I’d like to commend the authors for providing a detailed overview of the changes in Tanzania’s regulatory landscape.

**Major comments**

1. The sections summarizing major challenges are not supported by any references and it is not clear how these challenges were identified. Were there systematic efforts to assess how the regulatory system works, e.g. regular assessments of regulatory resources and outcomes or surveys of stakeholders’ perceptions ? This seems to be suggested in the discussion section. IT’s important to mention this in the results and to show that there was a feedback loop informing further reforms. Also, some of the major challenges have been not addressed by subsequent reforms. This should be also noted among the results.
2. The current structure (historical context, major challenges, how challenges were addressed) works quite well but there is a lot of repetition which could be reduced by re-organising the material. E.g., lack of independence and autonomy included under ‘Medicines regulatory capacity’ but to a certain extent already dealt with in the previous section ‘Medicines regulatory framework’. Paragraphs in the section ‘ Medicines promotion and advertising’ overlap with those in the section ‘Market surveillance and control.’
3. EAC regional harmonization is mentioned but it is not clear how this initiative impacted regulatory capacities in Tanzania. The aim of the harmonization is to use regulatory resources more efficiently through workload sharing, e.g. joint inspections. Similarly, other initiatives such as WHO collaborative registration procedure aim to reduce duplication and bring information sharing which is important for regulatory strengthening.

**Specific comments**

Abstract, Methods – The first sentence can be removed as the second one provides the information. Please delete the two references (51, 52). Please add information on analysis.

Abstract, Results – ‘improvement’ instead of ‘improved’ … a few more typos in this section, please edit.

Introduction, 5^th^ paragraph – vaccine plants mentioned here; in the methods, it’s stated that no vaccines are produced in Tanzania; the numbers provided in this paragraph need to be supported by references

Table 1 – please explain all abbreviations; columns 2-4 show institutions responsible for the specific regulatory functions; a more explanatory title would be useful e.g. ? Institutions and their regulatory functions in Tanzania, 1978 - 2020

Introduction, 10^th^ paragraph (“ The latter is responsible…” ) – “the remarkable improvements in regulatory system” what are these? Can this be supported by references?

Methods, 1^st^ paragraph – no need to repeat ‘1978 to 2020’ so many times.

Analytical framework, paragraph after [Insert Table 2] – the regulatory functions listed here slightly differ from those in Table to and Figure 1. Please ensure consistency.

Data sources and collection – make sure that all the references are correct. E.g. ref no. 53 says ‘updated 2021’ but the webpage currently lists two guidelines from 2020 and two published in 2022. It’s not clear which of these were analysed. It might be good to state your cut off date for the document analysis.

Table 3 – needs a more explanatory title e.g. overview of the key changes in laws, policies etc (?)

Medicines regulatory framework, major challenges – a lot of the material sounds more like historical context. Should this be moved to the previous sub-section?

Medicines regulatory framework, How challenges were addressed – multiple assessments are mentioned – could you provide more details and references. This seems to be the key to the improvements – analyses of what works and what doesn’t work and negotiations to bring in corrections.

Table 4 is great

Medicines regulatory capacity, how challenges were addressed – “in the batch of act” acts plural ? but looking at the references it seems to be one act?

Abbreviation DG used here and not explained; Registrar mentioned here – referring to the Pharmacy Council (?) … “foster collaboration between these agencies” Which agencies? Only TFDA mentioned in this paragraph.

The information on the TFDA annual budget in 2015/16 is missing

Medicines regulatory functions, historical context – no need to spell out the big numbers and please provide references for the data presented;

“In addition, the quality of the registration process is more thorough …” How? What is the evidence to support this claim?

Vigilance, major challenges – this paragraph needs some editing

Market surveillance and control, historical context – ‘National Essential Medicines List’ introduced previously under a different name

Market surveillance and control, major challenges, 2^nd^ paragraph needs to be developed

**General comments**

Regulatory effectiveness means that the regulation is effective in safeguarding the public and that the rules are enforced. The manuscript, however, primarily focuses on whether the appropriate structures, laws and rules are in place rather than effective enforcement and working leading to better functioning pharmaceutical sector.

There’s a lot of abbreviations used in the manuscript. Please make sure that they are all explained when introduced and consider adding a list of abbreviations at the end. Came across one abbreviation used for two different guidelines – GSDP – Good Supply and good distribution practices AND Good storage and distribution practices; the latter is correct.

Funding numbers in the text – 1.7 billion instead of 1,745,000,000 etc.

Hope this helps.
