# Supplementary material for: Strengthening regulation for medical products in Tanzania: An assessment of regulatory capacity development, 1978–2020
Source: PLOS Glob Public Health. 2024 Oct 16;4(10):e0003241. doi: 10.1371/journal.pgph.0003241 (PMC11482716; doi:10.1371/journal.pgph.0003241)
Supplement: Attachment — Submitted filename: 2024-05-06 Response to Reviewers PGPH-D-24-00071.docx [file pgph.0003241.s002.docx]

PLOS Global Public Health

Strengthening Regulation for Medical Products in Tanzania: An assessment of Regulatory Capacity Development, 1978–2020

**Responses to Editor’s**

**Major comments**

1. The sections summarizing major challenges are not supported by any references and it is not clear how these challenges were identified. Were there systematic efforts to assess how the regulatory system works, e.g. regular assessments of regulatory resources and outcomes or surveys of stakeholders’ perceptions ? This seems to be suggested in the discussion section. IT’s important to mention this in the results and to show that there was a feedback loop informing further reforms. Also, some of the major challenges have been not addressed by subsequent reforms. This should be also noted among the results

**Authors’ response:** We appreciate your feedback. The manuscript now includes cited references for the challenges and details on the systematic assessments used to identify them. We've clarified the feedback loop's impact on reforms in the results and acknowledged unaddressed challenges, enhancing the paper's clarity and depth based on your valuable insights.

2. The current structure (historical context, major challenges, how challenges were addressed) works quite well but there is a lot of repetition which could be reduced by re-organising the material. E.g., lack of independence and autonomy included under ‘Medicines regulatory capacity’ but to a certain extent already dealt with in the previous section ‘Medicines regulatory framework’. Paragraphs in the section ‘ Medicines promotion and advertising’ overlap with those in the section ‘Market surveillance and control.’

**Authors’ response:** **We appreciate your feedback. The replicative content has been removed** and retained only in the first section to appear.

3. EAC regional harmonization is mentioned but it is not clear how this initiative impacted regulatory capacities in Tanzania. The aim of the harmonization is to use regulatory resources more efficiently through workload sharing, e.g. joint inspections. Similarly, other initiatives such as WHO collaborative registration procedure aim to reduce duplication and bring information sharing which is important for regulatory strengthening.

**Authors’ response:** We appreciate the remark, however the paper did not go into harmonization as it is a big subject hard to combine here so we considered it outside of the scope of this manuscript. On the other the benefit is long term, and the process is at different stages progressing it is a bit premature to start commenting.

**Specific comments**

Abstract, Methods – The first sentence can be removed as the second one provides the information.

**Authors’ response:** The first sentence was deleted, and reviewer’s comment appreciated.

Please delete the two references (51, 52). Please add information on analysis.

**Authors’ response:** two references were deleted as suggested and reviewer’s comment appreciated.

Abstract, Results – ‘improvement’ instead of ‘improved’ … a few more typos in this section, please edit.

**Authors’ response: The** reviewer’s suggestion has been incorporated and comment appreciated.

Introduction, 5^th^ paragraph – vaccine plants mentioned here; in the methods, it’s stated that no vaccines are produced in Tanzania; the numbers provided in this paragraph need to be supported by references

**Authors’ response: The** reviewer’s suggestion has been incorporated three additional citations were added, comment appreciated.

Table 1 – please explain all abbreviations; columns 2-4 show institutions responsible for the specific regulatory functions; a more explanatory title would be useful e.g. ? Institutions and their regulatory functions in Tanzania, 1978 - 2020

**Authors’ response:** The reviewer’s suggestion has been incorporated the caption has been revised and all abbreviations have been explained

Introduction, 10^th^ paragraph (“ The latter is responsible…” ) – “the remarkable improvements in regulatory system” what are these? Can this be supported by references?

**Authors’ response:** The paragraph has been revised to include the appropriate citation indicating that TMDA is the first African regulatory agency to attain WHO Maturity Level 3. We acknowledge the reviewer's comment.

Methods, 1^st^ paragraph – no need to repeat ‘1978 to 2020’ so many times.

**Authors’ response:** The paragraph has been revised according to the reviewer's comment, ensuring repetitive wording is eliminated and maintaining "the 1978 to 2020’ " at one point at the end of the paragraph. The reviewer's input is highly appreciated.

Analytical framework, paragraph after [Insert Table 2] – the regulatory functions listed here slightly differ from those in Table to and Figure 1. Please ensure consistency.

**Authors’ response:** Yes, the opening paragraph reflects the existing standard according to the WHO Global Benchmarking Tool, which serves as a guideline for what is expected. The reviewer's comment has been acknowledged, indicating that these are the results of on-the-ground observations. There is a noted difference between the established standard and actual practice, suggesting a need for consistency. We recommend maintaining this distinction to help readers understand the gap between the WHO's expectations and what is observed in reality. By illustrating this inconsistency, readers can better appreciate the challenges and areas for improvement.

Data sources and collection – make sure that all the references are correct. E.g. ref no. 53 says ‘updated 2021’ but the webpage currently lists two guidelines from 2020 and two published in 2022. It’s not clear which of these were analysed. It might be good to state your cut off date for the document analysis.

**Authors’ response:.** The wording updated in 2021 has been corrected appropriately and now they list their reflects when the resource material was accessed and did not update which I think was misleading, cutoff date was the date the document/web was accessed for the document analysis. The reviewers comment is highly acknowledged

Revise Table 3 – needs a more explanatory title e.g. overview of the key changes in laws, policies etc (?)

**Authors’ response:.** We acknowledge constructive comments given by reviewer and now the title of the table has been revised is advised. The new title now reads ‘’Table 3: Overview of Key Changes in Medicines Regulatory Framework – Summary of Findings’’

Medicines regulatory framework, major challenges – a lot of the material sounds more like historical context. Should this be moved to the previous sub-section?

Medicines regulatory framework, How challenges were addressed – multiple assessments are mentioned – could you provide more details and references. This seems to be the key to the improvements – analyses of what works and what doesn’t work and negotiations to bring in corrections.

**Authors’ response:.** On moving the material in the previous sub section was not clear we have addressed the second comment of providing more details and references to the same. Reviewers comment is highly acknowledged.

Table 4 is great

**Authors’ response:.** Thank you

Medicines regulatory capacity, how challenges were addressed – “in the batch of act” acts plural ? but looking at the references it seems to be one act?

**Authors’ response:.** We acknowledge comments, correction has been made as suggested.

Abbreviation DG used here and not explained; Registrar mentioned here – referring to the Pharmacy Council (?) … “foster collaboration between these agencies” Which agencies? Only TFDA mentioned in this paragraph.

**Authors’ response:** We acknowledge comments, The abbreviation has been explained, subset sentence has been qualified to read However, setting of fees and charges for services are not determined by the TFDA-DG or Pharmacy council Registrar.

The information on the TFDA annual budget in 2015/16 is missing

**Authors’ response:** The missing information has been added, and has been corrected to reflected exactly amount, reviewers comments are highly acknowledged.

Medicines regulatory functions, historical context – no need to spell out the big numbers and please provide references for the data presented;

**Authors’ response:** Big numbers removed as suggested by reviewers and also references provided, reviewers comments are highly acknowledged

“In addition, the quality of the registration process is more thorough …” How? What is the evidence to support this claim?

**Authors’ response:** . A citation has been added in addition TMDA attained WHO Maturity Level 3 in 2018. A critical function assessed by WHO is Registration and Marketing Authorization. GBT Indicators for MA function include indicators on adoption/implementation of guidelines on requirements for demonstration of quality, safety and efficacy which are based on international standards (CTD). WHO assessors also reviewed the competency of assessors and completeness and depth of the assessment reports. References: WHO GBT, WHO Webpage listing TMDA as ML3 Agency [WHO. List of National Regulatory Authorities (Nras) Operating at Maturity Level 3 (Ml3)1 and Maturity Level 4 (Ml4)2 (as Benchmarked against Who Global Benchmarking Tool (Gbt) 2024 [Available from: https://cdn.who.int/media/docs/default-source/medicines/regulatory-systems/wla/list-of-nras-operating-at-ml3-and-ml4.v2.pdf?sfvrsn=ee93064f_14&download=true.]

Vigilance, major challenges – this paragraph needs some editing.

**Authors’ response:.** Indeed, there was no clarity on this paragraph reviewers comment has been considered and the entire paragraph has been rewritten to make it more clear and they added some references. reviewer's comment is highly acknowledged.

Market surveillance and control, historical context – ‘National Essential Medicines List’ introduced previously under a different name

**Authors’ response:.** It was not clear what did the previous different name was as it appears thas National Essential Medicines List is consistently use in the entire manuscript.

Market surveillance and control, major challenges, 2^nd^ paragraph needs to be developed

**Authors’ response.** The paragraph has been further developed and revised as suggested by the reviewer, whose comments are highly appreciated.

**General comments**

Regulatory effectiveness means that the regulation is effective in safeguarding the public and that the rules are enforced. The manuscript, however, primarily focuses on whether the appropriate structures, laws and rules are in place rather than effective enforcement and working leading to better functioning pharmaceutical sector.

**Authors’ response.** Reviewers’ comments are highly acknowledged we have revised where appropriate to replace the word regulatory effectiveness with the existence of appropriate structures for well-functioning of the regulatory system.

There’s a lot of abbreviations used in the manuscript. Please make sure that they are all explained when introduced and consider adding a list of abbreviations at the end. Came across one abbreviation used for two different guidelines – GSDP – Good Supply and good distribution practices AND Good storage and distribution practices; the latter is correct.

**Authors’ response.** The long form of the abbreviation has been corrected Andy we have checked the with the rest of the manuscript to ensure that the abbreviation is made at the first dimension in the months script subsequent mention it only the abbreviation is used and not the whole world. Reviewer comments are highly acknowledged.

Funding numbers in the text – 1.7 billion instead of 1,745,000,000 etc.

Hope this helps.

**Authors’ response. All the monetary big figures were rounded in light of the reviewers comment the reviewers’ comments are highly acknowledged.**

4. **Review Comments to the Author**

**Responses to Reviewers comments r’s**

Please use the space provided to explain your answers to the questions above. You may also include additional comments for the author, including concerns about dual publication, research ethics, or publication ethics. (Please upload your review as an attachment if it exceeds 20,000 characters)

**Reviewer #1:** Thank you for the opportunity to comment on this interesting paper. I’d like to commend the authors for providing a detailed overview of the changes in Tanzania’s regulatory landscape.

**Authors’ response. Thank you**

Major comments
1. The sections summarizing major challenges are not supported by any references and it is not clear how these challenges were identified. Were there systematic efforts to assess how the regulatory system works, e.g. regular assessments of regulatory resources and outcomes or surveys of stakeholders’ perceptions ? This seems to be suggested in the discussion section. IT’s important to mention this in the results and to show that there was a feedback loop informing further reforms. Also, some of the major challenges have been not addressed by subsequent reforms. This should be also noted among the results.

**Authors’ response.** The data sources and collection methods were described in earlier sections, with specific citations for each. The focus of this study, "Strengthening Regulation for Medical Products in Tanzania: An Assessment of Regulatory Capacity Development," was to evaluate regulatory capacity development and the contributions of various stakeholders.

While the reviewer's suggestion to include outcomes or surveys of stakeholder perceptions is valid, it falls outside the scope of this study. A statement ….. *It is important to note that not all challenges were addressed by these reforms and improvement is continuous.* Nevertheless, the reviewer's feedback is highly appreciated. We acknowledge its potential for guiding future research or follow-up studies that could explore stakeholder perceptions in greater detail**.**

2. The current structure (historical context, major challenges, how challenges were addressed) works quite well but there is a lot of repetition which could be reduced by re-organising the material. E.g., lack of independence and autonomy included under ‘Medicines regulatory capacity’ but to a certain extent already dealt with in the previous section ‘Medicines regulatory framework’. Paragraphs in the section ‘ Medicines promotion and advertising’ overlap with those in the section ‘Market surveillance and control.’

**Authors’ response:** We appreciate your feedback the replicative content has been removed and retained only in the first section to appear.

3. EAC regional harmonization is mentioned but it is not clear how this initiative impacted regulatory capacities in Tanzania. The aim of the harmonization is to use regulatory resources more efficiently through workload sharing, e.g. joint inspections. Similarly, other initiatives such as WHO collaborative registration procedure aim to reduce duplication and bring information sharing which is important for regulatory strengthening.
**Authors’ response:** We appreciate reviewer comments are highly acknowledged however harmonization and other initiatives such as WHO collaborative registration procedure remain outside the scope of this present study.

please see the attached file for more detailed comments

Reviewer #2: Thank you for the opportunity to review the attached “Strengthening Regulation for Medical Products in Tanzania: An Assessment of Regulatory Capacity Development, 1978–2020”. I found this piece detailing the evolution of regulatory capacity in Tanzania very interesting and worthwhile, although there are significant issues that I recommend need to be addressed prior to being considered for publication.

Firstly, I encountered difficulty reconciling the objectives outlined in the abstract and introductory sections with the actual content and main findings of the article. For instance, the abstract stated that ”we analyse changes in the regulation of the Tanzania pharmaceutical sector over three main periods that map to points of significant change in the sector “; and then, “The study identified three key periods of transformation in Tanzania's pharmaceutical regulation: 1) The separation of medicines regulation from food safety (1978 to 2003), 2) The expansion of regulatory domains and the establishment of a semi-autonomous regulatory agency (2003–2011), and 3) The expanded role of the Pharmacy Council, including premise regulation (2011 to 2020)”. However, these three key periods have not been explicitly developed elsewhere. While the evolution of the legal framework implies the existence of these phases, a comprehensive elaboration of how changes to the regulatory framework, regulatory capacities, and core functions vary throughout each period is warranted in the results section. Similarly, the information presented in Tables 1, 3, and 4 is inconsistent with these three temporal stages highlighted. Furthermore, the authors stated, “This led us to ask how such capacity was developed and whether that history held implications for other countries wishing to strengthen their own medicines regulatory capacity.” However, the article neglects to delve into the mechanisms and rationale behind how the Tanzanian regulatory system's evolution and effectiveness could serve as a model for informing policy to strengthen regulatory frameworks in similar contexts.

**Authors’ response:** We have carefully gone over the texts mentioned and where it was possible we implemented the reviewer’s suggestion.

Secondly, and the much more pressing issue, is the contribution of this study. I struggle to see how this research will significantly impact the literature on regulatory system strengthening beyond what has been previously documented. Notably, factors such as agency independence, accountability, and the influence of political, financial, and infrastructural elements have been extensively emphasized as pivotal for advancing toward an effective regulatory system (See, for example Shi, J., Chen, X., Hu, H., & Ung, C. O. L. (2023). Application of implementation science framework to develop and adopt regulatory science in different national regulatory authorities. Frontiers in Public Health, 11, 1172557; Regulatory System Strengthening in the Americas. Lessons Learned from the National Regulatory Authorities of Regional Reference. Washington, D.C.: Pan American Health Organization (2021); Chahal HS, Kashfipour F, Susko M, Feachem NS, Boyle C. Establishing a regulatory value chain model: An innovative approach to strengthening medicines regulatory systems in resource-constrained settings. Rev Panam Salud Publica. 2016;39(5):299–305).
Furthermore, the interpretation of results in the discussion section should encompass an analysis of how these findings augment or align with existing understandings of the issue. Given the article's focus on elucidating factors influencing the evolution of Tanzania's medicines regulatory authority, an examination from the perspective of institutional change theory could provide valuable insights (see: Coccia, Mario, An Introduction to the Theories of Institutional Change (2018). Journal of Economics Library, vol. 5, n. 4, pp. 337-344, 2018).
Finally, in response to “how such capacity was developed”, the identification of “3) Continuous capacity building and regulatory systems strengthening” in the discussion section, seems somewhat tautological to the research premise.

**Authors’ response:** While this subject has been covered by other authors, we believe our context is unique. Most other studies were conducted in developed countries like the United States, China, and similar nations, which differ significantly from developing countries in terms of resources and environment. We think the reviewer might have missed this key point.

Tanzania's context is unique, providing insights that other developing countries in Africa might find valuable. Tanzania was the first among the entire African continent nations to achieve WHO Maturity Level 3 in its regulatory system. This milestone demonstrates how Tanzania built its capacity, offering lessons for other countries with similar Geopolitical challenges. We respectfully disagree with the reviewer who suggested that this paper offers little to other nations; on the contrary, it can be a model for others seeking to improve their regulatory systems.

It is important to consider each country's specific context when comparing health systems. Imposing models from entirely different environments can be misleading. Despite our disagreement, we acknowledge the reviewer’s comments and will use them to further improve our manuscript.

Thirdly, there are significant issues concerning the structure of the article, as well as the methodological design and analytical framework, which warrant review:

- The structure and content of each section require careful review. While the introductory section initially outlines the significance of effective regulation and the challenges encountered in regulatory strengthening in sub-Saharan Africa, it also incorporates information that, in my view, pertains more to the findings.
For instance, “… They embarked on a series of capacity building interventions that included human resource capacity building, organizational systems strengthening, decentralization of operations, and the promotion of financial sustainability” …” The recorded change in improvement in regulatory system in Tanzania was not a standalone effort. Various development partners have contributed both technical assistance (TA) and financial assistance…”), seem to belong more to the findings section. Moreover, towards the end of the introduction, the authors state ,” The paper is organized as follows: In the next sections, we present a contextual background of the pharmaceutical sector in Tanzania, then we present the methods used in the study…”. However, instead of a dedicated subsection to contextualize the pharmaceutical sector in Tanzania, the introduction is followed by the Methodology section.
- From a methodological standpoint, the study would benefit from a thorough description of the processes involved in conducting the documentary analysis of government documents and scoping literature. This should include detailed explanations of the data retrieval and collection methods, the timeline followed, and the procedures for data extraction and analysis. Additionally, the authors should aim to acknowledge more comprehensively the limitations inherent in both the study design and the findings. On the other hand, there is a need for a clearer elaboration on how the analytical framework was developed. This would involve providing insights into the rationale behind the framework's design, its components, and how it was applied to analyze the data collected. For instance, the authors utilized the WHO's 2019 Global Benchmarking Tool, notwithstanding that a newer version was issued in 2021. However, they did not expound upon this choice or justify the criteria for considering certain core functions or their components as categories or subcategories for the analysis. Furthermore, there is a lack of explanation regarding how the new analytical framework incorporates key indicators for each core function, such as leadership and crisis management, transparency, accountability, and communication. Efforts should be directed towards refining and validating the analytical framework to effectively map the evolution of core elements of the national regulatory system (e.g., legal bases, standards, guides and procedures, resources, etc.) and its essential regulatory functions.

**Authors’ response:** The text that was previously part of the background section has been moved to the discussion section, as suggested. We also updated outdated references to align with the 2021 version of the WHO Global Benchmarking Tool. We greatly appreciate the reviewer's comments.

However, we respectfully disagree with the reviewer's remark about a perceived lack of explanation regarding how the new analytical framework incorporates key indicators for each core function. Addressing this would require us to go beyond the scope of our study objective, which is why we believe it should be explored in a different paper. Nonetheless, thank you for your feedback, and we will consider it for future research.

Some other issues the authors should consider as they rework the manuscript:

1) Findings: This is a comprehensive review of the evolution of Tanzania’s NRA, and there was an emphasis on distinguishing the development of legal instruments, regulatory capacities, and functions over three periods (i.e., 1978 to 2003; 2003 to 2011; 2011-2020). The authors could construct a narrative review to report their findings over each period. For instance, it is suggested that three sub-sections corresponding to each period contain different changes in each of the dimensions analyzed.

**Authors’ response:** We highly appreciate the reviewer's insightful comment and have incorporated the suggestion into our text. In response, we have constructed a narrative review that addresses the evolution of Tanzania's National Regulatory Authority (NRA) over the three specified periods (1978 to 2003; 2003 to 2011; 2011 to 2020). Each sub-section now covers the unique developments in legal instruments, regulatory capacities, and functions for the respective timeframe. Thank you for your constructive feedback, which has helped refine our study's structure and presentation. If further adjustments are needed, we are open to additional suggestions.

2) Tables and figures: As a general recommendation for the authors, please consider spelling out abbreviations at first mention in tables or consider defining the abbreviations in the table note or figure caption, respectively, even though the abbreviations have been previously defined in the main text.

**Authors’ response**: We highly appreciate the reviewer's comment and have taken the recommendation to heart. In response, we have ensured that abbreviations are spelled out at first mention in tables or defined in the table notes or figure captions, even if they have been previously explained in the main text. Thank you for your helpful suggestion, which has contributed to improving the clarity and accessibility of our content. If further adjustments are needed, we are open to incorporating additional recommendations

4.1 Table 1: In my opinion, table 1 is misplaced in the Introduction section as the reader has not been clearly presented with the regulatory functions, and it has not been explained at this point why the variations in regulatory oversight are reported across three different time periods. It seems better suited for the Findings sub-section. Also, the authors need to clarify better what the term “Regulatory Function” refers to in this table and how it differs from the regulatory functions presented in Table 2 and Table 5; and described in the Methodology section: “We defined regulatory functions to include the major responsibilities of a medicines regulatory authority as described by WHO 3 30 39 These include 1) National Regulatory Systems, 2) Medicines registration and marketing authorization, 3) vigilance, premises licensing, 4) regulatory inspection, 5) market surveillance and control, 6) clinical trials oversight, and 7) laboratory access and testing”. More suited terms for Table 1 might be “jurisdictional assignment” or “scope of regulatory oversight”.

**Authors’ response:** We highly appreciate the reviewer comment, and we wish to clarify that Table 1 highlighted the role of each regulator during those years. We have therefore renamed the content of Table 1 to read ‘’Role/function and not regulatory function.

4.2 Table 2: Heading “Analytic Categories” could be replaced by “Categories of analysis”.
Please include references in each category.

**Authors’ response:** We highly appreciate and agree with the reviewer’s comment. To make it clear, Table 2 has been simply renamed ‘’Overview of Key Changes in Medicines Regulatory Framework – Summary of Findings’’.
4.3 Table 3. It is unclear why this table only presents the results over two separate periods (i.e., 1978 to 2003; and 2003 to 2020) instead of three periods. This is confusing and makes it difficult for the reader to detect the key changes in the Tanzanian regulatory framework (e.g., The Pharmacy Act of 2002, the Tanzania Food, Drugs and Cosmetics Act of 2003, the Pharmacy Act of 2011). Additionally, please carefully review using “not existing” to describe the quality and risk management approach before the Tanzania Food, Drugs and Cosmetics Act of 2003 enactment, as it oversimplifies how (although incipient) quality assurance was regulated before the 2003 Act or subsequent regulations. Finally, please consider aligning the “Domains” described with those “Regulatory functions” outlined in Table 1. For instance, in Table 1 Retail and wholesale premises licensing falls under the Pharmacy Council from 2003 onwards, but this “domain” is not included in Table 3 under Pharmacy Council.

**Authors’ response:** We highly appreciate and agree with the reviewer’s comment. We have renamed Table 1 Institutions and their regulatory functions in Tanzania, in the three study periods between 1978 – 2020 We have also aligned the domain described in Table 1.

4.4 Table 4: Please see my comment above on using two instead of three periods to report the findings. New information such as the number of staff members and technology access not presented in the article body needs to include a reference or source of information. Also, please explain how “Low domestic production capacity,” -which seems to be a characteristic of the local pharmaceutical market, represented a challenge for the regulatory capacity between 2003 and 2020.

**Authors’ response:** We highly appreciate and agree with the reviewer’s comment. The issue of low production capacity is important to highlight a country relying on importation of over 90% need significant resources to ensure quality, safety and efficacy of imported medical products.

4.5. Table 6: Please add sources of information.
**Authors’ response:** We highly appreciate and agree with the reviewer’s comment and wish to clarify that highlighted information is sourced from TMDA.

3) Overall comment about grammar and style: there are many grammatical mistakes, and reading can be awkward due to sentence structure and style. Additionally, important references are missing. For example, the fifth paragraph of the introduction lacks proper citations for the information regarding the characteristics of the pharmaceutical sector; and the definition of categories Regulatory Framework and Regulatory Capacity lack references in both text and Table 2.
**Authors’ response:** We highly appreciate and agree with the reviewer’s comment. We have addressed all raised observations raised by the reviewers.

Again, thank you for the opportunity to review this manuscript. I hope my suggestions are useful to the authors.

Reviewer #3: This article explores the development of pharmaceutical regulatory capacity in Tanzania. It offers a clear organizational scheme and analytic structure, and lots of systematic evidence across analytic categories of interest to document its findings.

Some larger and smaller suggestions would enhance the analytic value and utility of the paper, as well as its clarity. I would encourage the authors to pursue them.

-- Larger suggestions

The analytic framing and implications could use some context and development. Here area a few questions and a few related pieces of scholarship to engage, all of which would help in these areas, particularly in the introduction and discussion/conclusion:

Do all national pharmaceutical regulatory bodies go through a similar process over time? What’s unique about this one? If yes, why, if not, why not?

**Authors’ response:** We highly appreciate and wish to clarify this may be true or yes. It is normally due to the need of the NRA to align itself with changes in science and technology, regional and international standards. This is the case for RFDA. Similarly, NDA is in the process of restructuring to align with the proposed regional model.

In what national regulatory body cases wouldn’t the components in the introduction and/or on page 22 obtain?

What makes the case of Tanzania unique in general, and/or in Africa?

**Authors’ response**: We highly appreciate reviewer's comment and wish to clarify that Tanzania is unique because it was the first country in Africa to have an NRA which is WHO ML3 in 2018. Currently, is among five ML3 NRA in Africa.

When would we not expect the kinds of improvements over time that we see here?

**Authors’ response:** We greatly value the reviewer's insightful question. Addressing it involves considering various factors, notably the unique circumstances of each country.

What’s the politics behind all of this, and at different points in time?

**Authors’ response:** The politics surrounding medical product regulation in Tanzania, as in many African countries, are subject to occasional interference. We acknowledge the reviewer's astute observation and value their inquiry into this complex aspect of our work

On the general framework and history, it’d be worth engaging a range of literature, all of which are described below.

First is Dan Carpenter’s book, including the chapter(s) (9? 11?) on comparative cases:

Carpenter, D., 2010. Reputation and power: Organizational image and pharmaceutical regulation at the FDA. In Reputation and Power. Princeton University Press.

On further comparative components, this edited book by Greer/King/Massard da Fonseca/Peralta-Santos would be helpful:

Greer, Scott L., Elizabeth King, Elize Massard da Fonseca, and Andre Peralta-Santos. Coronavirus politics: The comparative politics and policy of COVID-19. University of Michigan Press, 2021.

Likewise, a recent special issue of JHPPL edited by Jarman/Massard da Fonseca/King would be helpful in various ways:

<https://read.dukeupress.edu/jhppl/issue/49/1>

Particularly so the article by the Editors, and by Nachlis and Thomson, particularly on the conclusions and recommendations issue:

Jarman, Holly, Elize Massard da Fonseca, and Elizabeth J. King. "The Political Economy of Vaccines during the COVID-19 Pandemic." Journal of Health Politics, Policy and Law 49, no. 1 (2024): 1-8.

Nachlis, Herschel, and Kyle Thomson. "Emergency Regulatory Procedures, Pharmaceutical Regulatory Politics, and the Political Economy of Vaccine Regulation in the COVID-19 Pandemic." Journal of Health Politics, Policy and Law 49, no. 1 (2024): 73-98.

On the points about weaknesses in pharmaceutical regulatory structures amidst growing strengths, see the Nachlis article here:

Nachlis, Herschel. "Pockets of weakness in strong institutions: Post-marketing regulation, psychopharmaceutical drugs, and medical autonomy, 1938–1982." Studies in American Political Development 32, no. 2 (2018): 257-291.

**Authors' Response:** We are grateful to the reviewers for suggesting a deeper dive into the literature. To provide a broader framework and historical context, we incorporated references 36, 44, 45, and 46 into the background. This greatly enhanced the global perspective of our manuscript. We appreciate the reviewer's insightful question and the impact it had on improving our work.

-- Smaller suggestions

- Table 1 is confusing – the columns are labeled as years but describe organizations.

**Authors' Response:** We are grateful to the reviewers and the title has be revised to accommodate the suggestions. See Table 1: Institutions and their regulatory functions in Tanzania, in the three study periods between 1978 – 2020

- Table 6 – the numbers don’t add up to the total.

**Authors' Response:** The reviewers keen is acknowledge and the top raw figure

- Table 6 – was there really $8 billion in support? That seems high, but perhaps it is not.

**Authors' Response:** Yes, confirmed this is a cumulative figure from 2007-2020

- Figure 1 isn’t very clear, or at least the first 2/3 of it (the bottom 1/3 is more helpful).

**Authors' Response:** An Analytic Regulatory Framework refers to a structured approach or system used to analyze and interpret regulatory requirements within a specific context, such as a particular industry or jurisdiction. This framework typically involves breaking down regulatory guidelines, laws, and policies into manageable components for better understanding, interpretation, and compliance. It may include tools, and processes to assess the impact of regulations, identify gaps or inconsistencies, and develop strategies for effective regulatory implementation.

- The writing becomes redundant at times in a range of places, and these portions could be cut down to reduce length and enhance clarity.
- The analysis can be quite general at times in a range of places, and these portions could likewise be cut down to reduce length and enhance clarity.

**Authors' Response: Both** two points raised by reviewers suggestion acknowledge and effected at various locations
- Some of the descriptors are a bit confusing in some places (for example, “clamours” and “saddled”)

**Authors' Response:** The reviewers' suggestions have been acknowledged and implemented at various locations. We have also reviewed all confusing descriptors and replaced them with clearer terms

Again, overall, this is a very and very helpful article, with lots of good information, that could also benefit from all of the preceding suggestions.

**Authors' Response:** Thank you

**Additional checked in-house Comments**

1. Please provide separate figure files in .tif or .eps format only, and remove any figures embedded in your manuscript file. Please also ensure that all files are under our size limit of 10MB. You may leave the figure captions or legends in the manuscript.

Author Response: Three separate figures have been uploaded at tiff format

For more information about how to convert your figure files please see our guidelines: <https://journals.plos.org/globalpublichealth/s/figures>


2. We do not publish any copyright or trademark symbols that usually accompany proprietary names, eg (R), (C), or TM  (e.g. next to drug or reagent names). Please remove all instances of trademark/copyright symbols throughout the text, including ® on pages 13 and 45.

Author Response: editors comment acknowledge the and the affected the as appropriately
3. Please provide a detailed online Financial Disclosure statement. This is published with the article. It must therefore be completed in full sentences and contain the exact wording you wish to be published.

a) Please clarify all sources of financial support for your study. List the grants, grant numbers, and organizations that funded your study, including funding received from your institution. Please note that suppliers of material support, including research materials, should be recognized in the Acknowledgements section rather than in the Financial Disclosure.
b) State the initials, alongside each funding source, of each author to receive each grant. For example: "This work was supported by the National Institutes of Health (####### to AM; ###### to CJ) and the National Science Foundation (###### to AM)."
c) State what role the funders took in the study. If the funders had no role in your study, please state: “The funders had no role in study design, data collection and analysis, decision to publish, or preparation of the manuscript.”

If you did not receive any funding for this study, please simply state: “The authors received no specific funding for this work.”

Author Response: The authors received no specific funding for this work

4. Please ensure that the funders and grant numbers match between the Financial Disclosure field and the Funding Information tab in your submission form. Note that the funders must be provided in the same order in both places as well.

Author Response: The authors received no specific funding for this work
5. Please update your online Competing Interests statement. If you have no competing interests to declare, please state: “The authors have declared that no competing interests exist.”

Author Response: The authors have declared that no competing interests exist.
6. At this time, please confirm that your submission contains your "minimal data set", which PLOS defines as consisting of the data set used to reach the conclusions drawn in the manuscript with related metadata and methods, and any additional data required to replicate the reported study findings in their entirety. This includes:

a) The values behind the means, standard deviations and other measures reported;

b) The values used to build graphs;

c) The points extracted from images for analysis.

Author Response: We confirm that your submission contains your "minimal data set",
