# Supplementary material for: Strengthening regulation for medical products in Tanzania: An assessment of regulatory capacity development, 1978–2020
Source: PLOS Glob Public Health. 2024 Oct 16;4(10):e0003241. doi: 10.1371/journal.pgph.0003241 (PMC11482716; doi:10.1371/journal.pgph.0003241)
Supplement: Attachment — Submitted filename: Response to Reviewers-2024-09-06 .docx [file pgph.0003241.s003.docx]

PGPH-D-24-00071R1

Responses below

| SN | Editors Comments | Author Response: | Page changed |
| --- | --- | --- | --- |
| 1 | In the abstract and conclusion, you mention the importance of international partners such as the Global Fund, UNDP, and MSH. However, the analysis fails to explain how these organizations contributed to the process of strengthening regulation in Tanzania. | Reviewer’s comment is noted. However, we wish to clarify that the role of each development partner has been described under *Actively engaged community of stakeholders and international donors* | Pg.22 |
| 2 | As mentioned by R1 and R2, the response to the "so what" question has not been adequately addressed yet. What does this manuscript add to our knowledge about regulatory system strengthening? | Reviewer’s comment is noted. We wish to clarify that this manuscript contributes to the existing knowledge in that it is possible for other NRAs in Africa to achieve what TMDA achieved in 2018. It also shows that development partners can positively influence the implementation of WHO Global Benchmarking Tool (GBT). Similarly, documentation of achievement made by a particular NRA can be a catalyst to other NRAs. TMDA and other NRAs that have achieved ML3 status provide a platform where other NRAs visit and learn how TMDA did it. | NA |
| 3 | R1 still has some crucial comments that need to be addressed: better presentation of the evidence, formatting, and editing of the manuscript. The fact that there is a paragraph duplicated within the introduction suggests that the manuscript was not carefully revised. | Reviewer’s comment is noted and therefore the whole manuscript has been revised and corrections made where necessary. | Multiple |

Please note that this is your last chance to make the necessary revisions. Failure to adequately address these points will result in the rejection of your manuscript.

Please submit your revised manuscript by Aug 17 2024 11:59PM. If you will need more time than this to complete your revisions, please reply to this message or contact the journal office at [globalpubhealth@plos.org](mailto:globalpubhealth@plos.org). When you're ready to submit your revision, log on to <https://www.editorialmanager.com/pgph/> and select the 'Submissions Needing Revision' folder to locate your manuscript file.

Please include the following items when submitting your revised manuscript:

- A rebuttal letter that responds to each point raised by the editor and reviewer(s). You should upload this letter as a separate file labeled 'Response to Reviewers'.
- A marked-up copy of your manuscript that highlights changes made to the original version. You should upload this as a separate file labeled 'Revised Manuscript with Track Changes'.
- An unmarked version of your revised paper without tracked changes. You should upload this as a separate file labeled 'Manuscript'.

6. Review Comments to the Author

| SN | Reviewer #1Comments | Author Response: | Page changed |
| --- | --- | --- | --- |
| 1 | Abstract Background – ‘for well-functioning of the regulatory system’ ... why not “a well-functioning regulatory system” ? (here and later in the manuscript) | Thank you very much for your valuable feedback. I greatly appreciate your thorough review and have made the proposed changes as advised. Thank you once again for your insightful comments and guidance | Page 2&5 |
| 2 | Results, 1st sentence – there’s redundant ‘of’ before ‘the regulatory capacity | I have carefully considered your suggestion and have removed the redundant wording as advised. I appreciate your valuable insights and guidance | Pg 2 line 59 |
| 3 | Results, 2nd sentence – ‘under mirror’ ? do you mean ‘under the microscope’ ; the following ‘correspond to’ is redundant; ‘and’ before the point 2) should be moved to before the point 3) | Reviewer’s comment is noted. However, we wish to clarify that we meant the three periods under review. To avoid such confusion the word mirror has been changed to periods under review | Pg 2 line 61 |
| 4 | Conclusions – the first sentence need a full stop at the end and a space to be added before the 2nd sentence. Delete ‘, and others’ at the end of the 2nd para. | Reviewer’s comment is noted, and a full stop and a space has been introduced as recommended. | Pg.3 lines 71-73. |
| 5 | The 3rd para. Does not follow from the results presented (here and in the conclusion). | Reviewer's comment is noted, and the respective paragraph has been rephrased. | Pg. 3 lines 82-85 |
| 6 | Introduction Para.5 – ‘Effective medicine regulation’ ? was it effective or active? | Reviewers comment is acknowledged and the word effective has been now replaced you with the active. | See Pag 4 Lines 117 |
| 7 | Para. 6 – ‘as well as organizational structures’ ? under a different organizational structures; this part need a better formulation | the entire paragraph has been reformulated to make it clearer as suggested by reviewers. viewers comment is highly acknowledged | Page 4  Lines 126-129 |
| 8 | Para. 8 – ‘However’ is not the right word to use here. This paragraph refers to the WHO guidance and recommendations in this area without mentioning the WHO. The next para then starts with ‘using this framework’ but it’s not clear what framework is being referred to. | The sentence has been rephased ……Recently, a consensus has emerged regarding the essential functions necessary for active regulation., The subsequent paragraph has been rephrased too | Page 5  Line 141  Line 148 |
| 9 | Para. After “[insert table 1] – delete “In” and start with “A recent ...” In this sentence FDA is mentioned but the abbreviation is not explained. In the 2nd sentence it is not clear what “the latter” refers to. TO corona politics? | Abbreviation is defined at first appearance in abstract  The latter was referring to TMDA and now on the sentence has been rephrased to make it clearer. Reviewers comment is highly acknowledged | Page 2 Lines 41-42  Page 5  Lines 157-158 |
| 10 | The next para. Repeats the sentence on Carpenter’s chapter | It was not clear if the next paragraph referred is the one beginning …. The recorded change in improvement in regulatory system in Tanzania. If this is the case, I cannot link wit Carpenter’s chapter | NA |
| 11 | Penultimate paragraph – the outlined organization of the paper does not correspond to what follows |  |  |
| 12 | Methods, objectives - how do you assess the effectiveness of the regulatory system? | -The viewer’s comment is highly appreciative, acknowledging that the paragraph has been rephrased effectively. The effectiveness aspect has been removed, while the regulatory evolution has been retained. | Page 6  Line 186, |
| 13 | Analytical framework, 1st sentence – ‘our study objective’ should be plural ‘objectives’? | Reviewer's comment is noted revision has been effected as advice. | Page 6  Lines 196 |
| 14 | - 2nd para. It looks like that instead of regrouping you considered the above mentioned dimensions under regulatory functions and complemented with two other categories (re. Framework, reg. Capacity) | Reviewer's comment is noted and acknowledged | NA |
| 15 | Data sources and collection – ‘conducted key informant interviews’ - more details needed here on how many, when, focus of the interviews (structured, open end questions, etc), how analysed; ethical approval  The next sentence “The of data included...” needs correction | Reviewer is highly appreciated for valuable feedback. We sincerely apologize for the oversight in mentioning the key informant interviews. While these interviews were initially planned, they were not conducted, as the study later shifted its focus to extensive documentary reviews. As a result, this approach was not part of the final methodology, which also eliminated the need for any questionnaires or ethical clearance. The paragraph has been revised appropriately | Page 7  Lines 223 |
| 15 | p.8 Major challenges – “in both periods analysed” – the abstract refers to three periods and so on... | Reviewer's comment is noted revision has been implemented as advised replacing ‘both’ with ‘all’ | Page 8  Line 258 |
| 16 | The discussion primarily provides a summary findings but does not relate to the broader literature. Here, the comment by Reviewer 2 is relevant – what does this manuscript add to what we already know about regulatory system strengthening? | The reviewer's comment is noted. We wish to clarify that our manuscript highlights how other National Regulatory Authorities (NRAs) in Africa can replicate the success achieved by the Tanzania Medicines and Medical Devices Authority (TMDA) in 2018. It demonstrates that development partners can have a positive impact on the implementation of the WHO Global Benchmarking Tool (GBT). Additionally, documenting the achievements of a particular NRA can serve as a catalyst for other NRAs. Those NRAs that have attained ML3 status, including TMDA, provide a valuable learning platform for their counterparts, offering insights into successful strategies and practices | NA |
| 17 | Reviewer #2: The authors addressed the comments previously raised. Please still check the consistency of references. I detected this paragraph duplicated within the same section (introduction) and with different reference numbers: "In a recent book chapter by Carpenter explains how the FDA's reputation and power have inter played in shaping regulation36 and recent corona politics 37 39-41" | Thank you for your insightful comments and the opportunity to improve our manuscript. We appreciate Reviewer #2’s observations regarding the consistency of our references. We have thoroughly reviewed and corrected these inconsistencies to ensure accuracy and reliability throughout the document.  We acknowledge the point raised about the interplay between the FDA’s reputation and power in shaping regulations, as mentioned in the duplicated paragraph. This error has been rectified by removing the duplication and streamlining the reference numbers for clarity.  Regarding the comparison of regulatory environments between the United States and Tanzania, we maintain that the distinct local contexts, political environments, and resource availability significantly differentiate the regulatory challenges faced by NRAs in developing countries. The regulatory landscape in Tanzania and similar African settings is influenced markedly by these factors, making direct comparisons with the FDA less pertinent. Consequently, we argue that the achievements of the Tanzania Medicines and Medical Devices Authority (TMDA) provide more relevant and practical lessons for other African NRAs striving to improve their regulatory frameworks under similar constraints.  We believe that the example of TMDA offers invaluable insights that are specifically tailored to the realities faced by regulators in resource-limited settings, making it a more applicable model for our African colleagues to learn from and emulate.  Thank you once again for your constructive feedback, which has undoubtedly strengthened our manuscript. We look forward to any further suggestions you might have. | Page 38-45 |
